# Feasibility analysis of flexible bronchoscopy in conjunction with noninvasive ventilation for therapy of hypoxemic patients with Central Airway Obstruction: a retrospective study

**DOI:** 10.7717/peerj.8687

**Published:** 2020-04-08

**Authors:** Xiaoke Chen, Yiping Zhou, Haiqiong Yu, Yue Peng, Liping Xia, Nian Liu, Hairong Lin

**Affiliations:** 1 Department of Respiratory Medicine, The Eighth Affiliated Hospital, Sun Yat-sen University, Shenzhen, China; 2 Department of Anesthesia, The Eighth Affiliated Hospital, Sun Yat-sen University, Shenzhen, China

**Keywords:** Bronchoscopy

## Abstract

**Background:**

Interventional bronchoscopy for hypoxemic patients with central airway obstruction (CAO) is typically performed under general anesthesia. This approach poses remarkable challenge for both bronchoscopist and anesthesiologist. Noninvasive ventilation (NIV) during flexible bronchoscopy (FB) has been successfully used in hypoxemic patients, but rarely in the treatment of hypoxemic patients with CAO.

**Objective:**

To evaluate the feasibility of therapeutic FB assisted with NIV for therapy of hypoxemic patients with CAO.

**Method:**

Twenty-nine hypoxemic CAO patients treated with FB from December 2010 to May 2016 in our hospital were retrospectively reviewed, either aided with NIV under sedation (NIV group ) or through artificial airway under general anesthesia (control group). Interventional procedures included balloon dilation, electrocautery and argon plasma coagulation

**Result:**

Fifteen patients were enrolled in the NIV group and 14 in the control group. The success rate (93.3% VS 92.9%, *p* = 1.0), procedure time (60.5 ± 4.2 min VS 67.8 ± 5.6 min, *p* = 0.31) and oxygenation improvement between the two groups have no significant difference. Less reduction of systolic blood pressure and heart rate during procedure was observed in the NIV group. The NIV group showed shorter admission time before procedure than the control group (35.1 ± 4.6 h VS 55.6 ± 5.6 h, *p* < 0.01). In addition, procedure fee in the NIV group was significantly less than that in the control group (540.7 ± 62.8$ VS975.4 ± 69.5$, *p* < 0.0001).

**Conclusion:**

FB assisted with NIV is a safe, efficient and economic method for therapy of selected hypoxemic patients with CAO.

## Introduction

The syndrome of central airway obstruction(CAO), a common problem in medical and surgical settings (Murgu et al., 2016; Barros Casas et al., 2014), is generally defined as occlusion of >50% of the trachea, mainstem bronchi, bronchus intermedius, or a lobar bronchus. The incidence of this disorder is rising at present, possibly due to (1) the epidemic of lung cancer, which is typically diagnosed at an advanced stage (Guibert et al., 2016) and up to 30% of which causes CAO (Lin & Chung, 2016); (2) the increasing use of artificial airways (Barros Casas et al., 2014); (3) the epidemic of tuberculosis, which is still out of control in parts of the world including Africa and Asia (Dheda et al., 2017). Especially the epidemic of tracheobronchial tuberculosis, which is present in 10%–40% of active pulmonary tuberculosis (Smith, Schillaci & Sarlin, 1987), with involvement of the primary bronchi in 60%–95% of cases (Kashyap, Mohapatra & Saini, 2003).

Patients with hypoxemic CAO are typically compromised with a life-threatening airway obstruction. In recent decades, interventional bronchoscopy has become an effective treatment for central airway obstruction (Guibert et al., 2016). However, given that a flexible bronchoscop (FB) can occupy 10–15% of the normal tracheal lumen and decrease PaO_2_ by 10–20 mmHg (Payne, Goyal & Gupta, 1986), it is not recommended for excessively dyspneic patients without assisted ventilation (Zias et al., 2009). The current recommendation for interventional therapy of hypoxemic CAO is either by rigid bronchoscopy or by FB via artificial airway under general anesthesia (Du Rand et al., 2011; Ost et al., 2015; Dutau, Vandemoortele & Breen, 2013; Mahmood et al., 2015). However, therapeutic bronchoscopy under general anesthesia for critically ill patients is unavailable at many bronchoscopy centers, especially in developing countries. A survey has shown that both rigid bronchoscopy and FB under general anesthesia are seldom performed in China (Nie et al., 2012). Additionally, economic burdens arising from general anesthesia should not be ignored.

Are there any other convenient and practical airway management techniques for the interventional bronchoscopy of hypoxemic CAO? In several recent studies, NIV has been successfully applied during FB for BAL or ETI under topical anesthesia with or without sedation in hypoxemic patients (Murgu, Pecson & Colt, 2010; Cabrini et al., 2013; Korkmaz Ekren et al., 2016). To our knowledge, no research has studied the use of NIV during interventional FB in patients with hypoxemic CAO to date.

In the present study, we review the efficacy and security of the technique and compare it with those of interventional FB via ETI or laryngeal mask (LMA) under general anesthesia performed in our hospital. The results of this medical research are reported as follows.

### Method

This is a retrospective review approved by the Institutional Ethics Committee of the Eighth Affiliated Hospital of Sun Yat-sen University (Ref # 2016091001). Hypoxemic CAO patients who were treated with interventional flexible bronchoscopy in our hospital during the period between December 2010 and May 2016 were included. Verbal informed consent was provided by every participant or their legal representatives. The authors did not have access to information that could identify individual participants during or after data collection.

Inclusion criteria are (1) patients conform to the diagnostic criteria of hypoxemic CAO; (2) Blood gas analysis on admission shows that PaO2 < 60 mmHg and CO2 PaCO2 ≥50 mmHg; (3) >50 years old; (4) on admission, with severe dyspnea symptoms, such as cough, purulent sputum, shortness of breath, jugular vein distention, etc.; with increased respiratory function, such as chest abdominal contradictory movement or intercostal muscle contraction.

Exclusion criteria are (1) stent placement; (2) significantly enhanced computed tomography (CT) of the lesion (elevated CT value over 50); (3) obvious respiratory depression, apnea, or obstruction of upper respiratory tract; (4) unstable cardiovascular system function, including hypotension, arrhythmia, myocardial infarction, or serious hemodynamic instability, no response to liquid therapy and vasoactive drugs; (5) the secretion is thick and large, which may cause aspiration; (6) serious liver and kidney dysfunction; (7) obesity, craniofacial trauma, nasopharyngeal abnormalities, etc., which can not be treated by FB; (8) other contraindication of FB as previously described (Du Rand et al., 2013). The intervention was considered successful if at least 50% of airway patency was achieved (Mahmood et al., 2015). Twenty-nine patients met the above criteria.

### Preparation before procedures

Standard preoperative assessment was made for each patient, consisting of laboratory investigations (including coagulation parameters and arterial blood gas analysis), diagnostic FB and chest CT. Data of location, percentage and length of the lesion and condition of distal airway were all obtained for making a decision of interventional procedure. Patients’ oral intake, except for water, was refrained for 4 h in the NIV group and 8 h in the control group prior to the intervention. All patients had venous access before the procedures.

### Procedures

#### NIV group

Additional exclusion criteria were ETI patients and patients with contraindications for NIV, including reduced level of consciousness, recent facial or esophageal surgery, and inability to tolerate the face mask (Cabrini et al., 2013).

Patients were sent to the bronchoscopy center and initiated on NIV to allow acclimatization. Dezocine (2.5–5 mg) was given intravenously 10min before the examination while midazolam (1–3 mg IV) was given immediately prior to FB, according to age and body weight. 2% lidocaine was used as topical anesthesia. A swab with 1% ephedrine and 2% lidocaine was retained in one nostril, 5 min before the insertion of FB.

**Figure 1:**
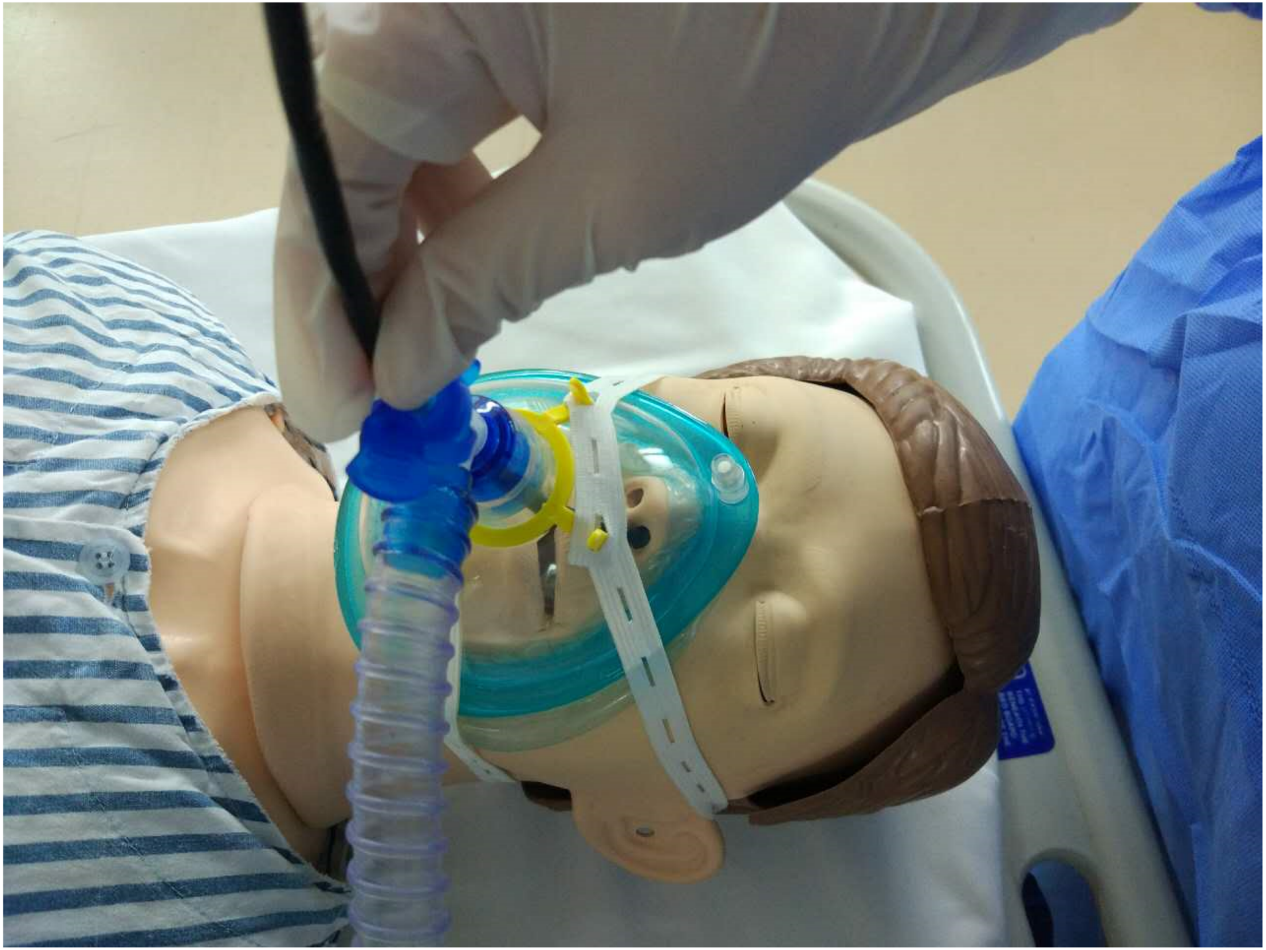
Therapeutic bronchoscopy during NIV. Flexible bronchoscope was introduced into the nose through the T-seal adapter and the full-face mask. The face mask was connected to a mechanical ventilator.

NIV mode of the ventilator (MAQUET SERVO) was delivered with positive endexpiratory pressure (PEEP) via a resuscitation full-face mask secured with headstraps (Fig. 1). PEEP was initially set at five cmH_2_O, pressure support at 10 cmH_2_O and FiO_2_ at 0.30. These parameters were adjusted during the procedures to make sure SpO_2_>90% and tidal volume>8 ml/kg. FiO_2_ was confirmed at 0.4 or below before thermal ablation to avoid airway fire.

FB (OlympusBF-1T40) was performed after the patients had received topical anesthesia. A T-adapter was attached to the face mask for insertion of FB via nasal route. If this initial insertion failed, a mouthpiece was placed for oral insertion to replace. Additional lidocaine (2%) was administered through the bronchoscope as needed for topical aneasthesia of the airway. Subsequent boluses of 1–2 mg midazolam were administered if the subject was not sufficiently sedated during the procedure.

Balloon Dilation, electrocautery needle /loop and APC via bronchoscope were selected solely or combined in accordance at the bronchoscopists discresion.

#### Control Group

Patients were sent to the operating room,and subsequently Anesthetized intravenously. The intravenous anesthesia included target controlled infusion (TCI) of propofol (2 ug/ml) and remifentanil (4–6 ng/ml) with Dexmedetomidine (0.5 ug/kg/h). The patients were ventilated by volume-controlled mode of anesthesia machine (Dräger GS) through ETI or LMA via a T-adapter (8.0 ETI for lower tracheal and bronchus lesions, 3–4#LMA for upper tracheal lesion). Tidal volume, was set at 6∼10 ml/kg, respiratory rate at 12∼16/min and oxygen flow at 5 litters. Oxygen and air flow were adjusted to ensure FiO_2_ at 0.4 or below just before thermal ablation to avoid airway fires.

### Monitoring

During the procedures, pulse-oximetry and heart rhythm (5-lead ECG) were continuously monitored. For NIV group, automated noninvasive blood pressure was monitored, while for control group, an arterial catheter was inserted into radial artery for blood withdrawal and hemodynamic monitoring.

### Statistical analysis

All statistical analysis was done with GraphPad Prism4 software (GraphPad Software Inc, La Jolla, CA, USA). Numeric and categorical parameters were analyzed by Fisher exact test. Differences of measurement data were presented as mean ±  standard deviation (SD) and were analyzed by t test for two groups or one-way analysis of variance for multiple groups. Mann–Whitney U test was used for analysis of admission hours before procedure and procedure fee. A *p*-value < 0.05 was considered statistically significant.

## Result

A total of 29 patients were enrolled in this study: 15 in the NIV group and 14 in the control group. Subject characteristics of both groups were comparable based on age, gender, and concomitant diseases, including hypertension, coronary heart disease and COPD. There was no significant difference between the two groups in lesion characteristics, including malignant rate, elevated CT value and site of obstruction (Table 1).

**Table 1:** Characteristics of patients and lesions.

	**NIV group (*n* = 15)**	**Control group (*n* = 14)**	***P* value**
**Male (%)**	10	10	1.00
**Age (mean ± SD)**	57.5 ± 15.1	61.6 ± 12.5	0.14
**Malignant (%)**	10	10	1.00
**Rise of enhanced CT value**	33.1 ± 10.1	35.1 ± 11.9	0.63
**Site of obstruction**			
**trachea(%)**	7	6	1.00
**bronchus(%)**	8	8	1.00
**Hypertension**	4	4	1.00
**Coronary heart disease(%)**	2	2	1.00
**COPD**	4	3	1.00

There was no significant difference in the type of procedures, procedure time and success rate between the two groups. The lowest SpO_2_ during procedure and improvement of PaO_2_ in the NIV group were similar to those in the control group (Table 2).

**Table 2:** Type of procedures, procedure time, changes of oxygenation parameters.

	**NIV group**	**Control group**	***P* value**
**Type of procedures**			
Apc	12	10	0.68
Cryotherapy	13	10	0.39
Balloon Dilation	5	4	1.00
Electrocautery	2	3	0.65
**Procedure time** (min)	60.5 ± 4.2	67.8 ± 5.6	0.31
**Success rate (%)**	100.0	100.0	1.00
**Lowest SPO2 during procedure**	92.4 ± 0.7	92.9 ± 0.5	0.53
**PaO2**			
before procedure	66.7 ± 1.9	67.3 ± 1.8	0.83
after procedure	79.9 ± 2.1	80.3 ± 2.3	0.90

**Figure 2:**
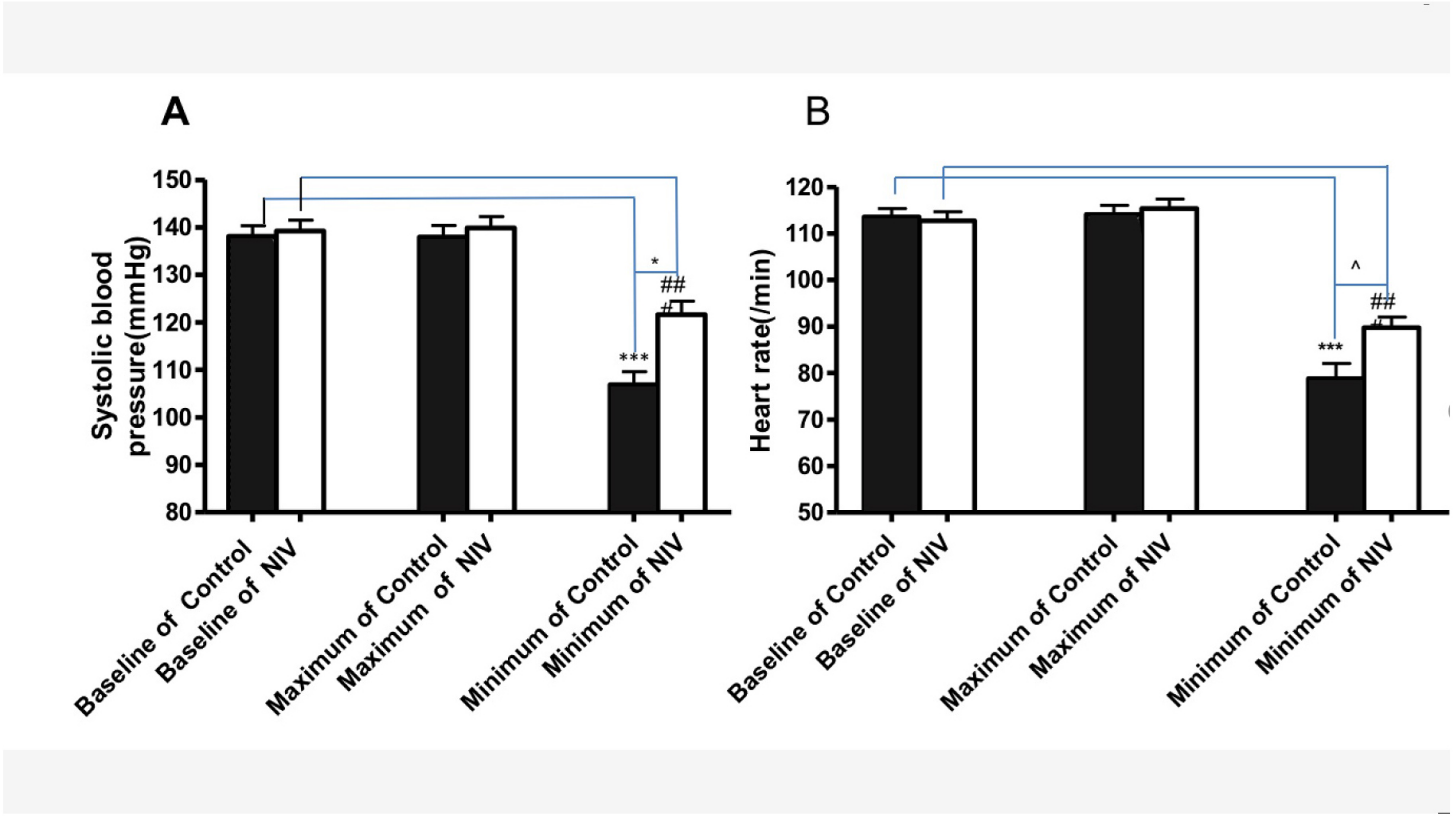
Changes of systolic blood pressure (A) and heart rate (B) during the procedure in NIV and control groups. ***p < 0.001 versus baseline of Control, ###*p* < 0.001 versus baseline of NIV; * *p* < 0.05 versus minimum systolic blood pressure of control; ^*p*^ < 0.001 versus minimum heart rate of control.

**Figure 3:**
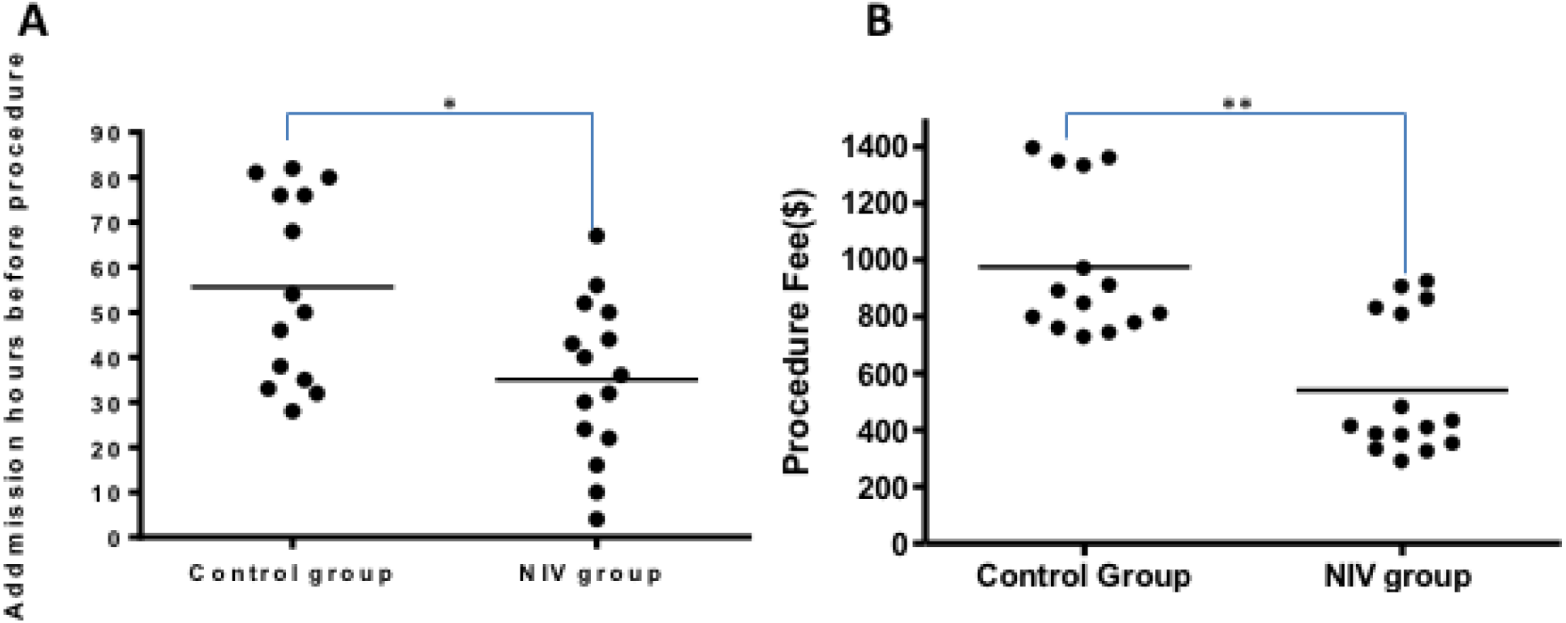
Admission hours before procedures and procedure fee (B) in NIV group and the control group. **p < 0.01 versus the control group, ***p < 0.001 versus the control group.

Less reduction of systolic blood pressure and heart rate during procedure was observed in the NIV group (Fig. 2). Compared with the control group, the NIV group showed less admission hours before procedure (35.1 ± 4.6 h VS 55.6 ± 5.6 h, *p* < 0.01), Fig. 3A. Additionally, procedure fee in the NIV group was significantly less than that of the control group (540.7 ± 62.8$ VS975.4 ± 69.5$, *p* < 0.0001), Fig. 3B.

## Discussion

The present study shows that the use of NIV is both safe and cost-effective in the treatment of hypoxemic patients with CAO.

Safety is the top concern of interventional therapy for hypoxemic CAO. There is no significant difference in SpO_2_ between the two groups during procedures. All the procedures were successfully performed. However, the suppression of blood pressure and heart rate is less in the NIV group than that in the control group. FiO_2_ and PEEP can be adjusted with NIV to ensure oxygenation, and inspiratory pressure can also be adjusted to ensure adequate ventilation. In case of longer procedure duration, Midazolam and dezocine, which were effective in our previous diagnostic bronchoscopy research (Chen et al., 2015), are used at relatively higher doses to reduce discomforts and ensure smooth procedures. As sedatives have less impact on respiratory and circulatory system than general anesthesia, less suppression of blood pressure and heart rate is observed in the NIV group.

As bronchoscopy for the control group is performed through an artificial airway under general anesthesia in the operation room, the patients and the interventional equipment in the control group have to be moved to operation room for the procedures. Sometimes the procedures are delayed due to the unavailability of operation room or skillful anesthesiologist. In contrast, procedures to the patients in the NIV group are performed without anesthesiologist in the bronchoscopy room. The procedures are carried out during NIV with normal adapter via resuscitation mask under moderate sedation. All the instruments, including NIV machine, adaptor and mask, are easy-to-get and equipped in the bronchoscopy room. As we Chinese pulmonologists are familiar with NIV and sedation techniques, the procedures are convenient and smooth.

The procedure duration of the control group is similar to what was described in the previous reports, from 67 min to 87 min (Vorasubin et al., 2014; Espinoza et al., 2015). Although it is not as easy to draw out and insert the bronchoscopy repeatedly without artificial airway in the NIV group as in the control group, the former achieves similar success rate as the latter, with no more procedure time. Possible reasons are as follows: (1) repeated insertions of bronchoscopy are maximally avoided with improved skills: tumors are destroyed by APC, then aspirated through working channel; massive necrosis is drawn out by cryotherapy after APC; and electrocautery loops are frequently used. (2) Moderate sedation reduces the discomfort of the patients and their vocal cords commonly stay open for the insertion of FB.

As it may be hard to handle massive hemoptysive under NIV, significant CT enhanced lesions are excluded in the study. It has been shown that both the incidence and mortality of massive bleeding induced by therapeutic bronchoscopies are significantly higher than those by diagnostic bronchoscopies (Zhou et al., 2016). In the case of hypoxemic CAO, such mortality will be even higher. Although there is no case with bleeding over 100 ml in the study, interventional pulmonologists should be skilled in the management of massive bleeding as well as CAO (Sakr & Dutau, 2010). Given that it is typically hard to insert bronchoscopy-aided intubation when the airway is full of blood in emergency, the operators are best to be skilled at ETI with laryngoscopy to manage more difficult airways.

As mentioned above, the procedures in the control group sometimes are delayed. Therefore, the admission hour before procedure in the NIV group is significantly shorter than that in the control group. The cost for the procedures in the NIV group, without general anesthesia and the involvement of anesthesiologist, is also significantly lower than that in the control group.

This study has some limitations. First, it is a retrospective study. Second, due to the limitation of case number in our hospital and the lack of medical records of some patients, the number of patients included in this study is small, and the reliability of the conclusions is weak. The number of samples needs to be further expanded to increase the credibility of this study. Third, procedures on most patients in the control group were performed prior to those in the NIV group, introducing the possibility of increased operation experience in the NIV group. Fourth, stent placements were excluded as it is hard to place airway stent with FB under NIV. Fifth, significant CT enhanced lesions are excluded in the study to avoid massive hemoptysis which is hard to manage in study group.

## Conclusions

In conclusion, the use of NIV with moderate anesthesia may be a safe and cost-effective procedure for non-stent therapeutic brochoscopy in treatment of hypoxemic CAO patients with: mild to moderate CT enchanced lesions. More perspective studies with larger sample sizes are required to make a more thorough assessment of the technique.

##  Supplemental Information

10.7717/peerj.8687/supp-1 Data S1 Raw data

## Additional Information and Declarations

### Data Availability

The following information was supplied regarding data availability: The raw data is available in the Supplemental Files.

## References

[ref-1] Barros Casas D, Fernández-Bussy S, Folch E, Flandes Aldeyturriaga J, Majid A (2014). Non-malignant central airway obstruction. Archivos de bronconeumología.

[ref-2] Cabrini L, Nobile L, Cama E, Borghi G, Pieri M, Bocchino S, Zangrillo A (2013). Non-invasive ventilation during upper endoscopies in adult patients. A systematic review. Minerva Anestesiologica.

[ref-3] Chen XK, Zhou YP, Zhang X, Xia LP, Li AF, Liu H, Yu HQ (2015). Conscious sedation with midazolam and dezocine for diagnostic flexible bronchoscopy. European Review for Medical and Pharmacological Sciences.

[ref-4] Dheda K, Gumbo T, Maartens G, Dooley KE, McNerney R, Murray M, Furin J, Nardell EA, London L, Lessem E, Theron G, Van Helden P, Niemann S, Merker M, Dowdy D, Van Rie A, Siu GK, Pasipanodya JG, Rodrigues C, Clark TG, Sirgel FA, Esmail A, Lin HH, Atre SR, Schaaf HS, Chang KC, Lange C, Nahid P, Udwadia ZF, Horsburgh CR, Churchyard GJ, Menzies D, Hesseling AC, Nuermberger E, McIlleron H, Fennelly KP, Goemaere E, Jaramillo E, Low M, Jara CM, Padayatchi N, Warren RM (2017). The epidemiology, pathogenesis, transmission, diagnosis, and management of multidrug-resistant, extensively drug-resistant, and incurable tuberculosis. The Lancet. Respiratory Medicine.

[ref-5] Du Rand IA, Barber PV, Goldring J, Lewis RA, Mandal S, Munavvar M, Rintoul RC, Shah PL, Singh S, Slade MG, Woolley A, Group BTSIBG (2011). British Thoracic Society guideline for advanced diagnostic and therapeutic flexible bronchoscopy in adults. Thorax.

[ref-6] Du Rand IA, Blaikley J, Booton R, Chaudhuri N, Gupta V, Khalid S, Mandal S, Martin J, Mills J, Navani N, Rahman NM, Wrightson JM, Munavvar M, Group BTSBG (2013). British Thoracic Society guideline for diagnostic flexible bronchoscopy in adults: accredited by NICE. Thorax.

[ref-7] Dutau H, Vandemoortele T, Breen DP (2013). Rigid bronchoscopy. Clinics in Chest Medicine.

[ref-8] Espinoza A, Neumann K, Halvorsen PS, Sundset A, Kongerud J, Fosse E (2015). Critical airway obstruction: challenges in airway management and ven tilation during therapeutic bronchoscopy. Journal of Bronchology & Interventional Pulmonology.

[ref-9] Guibert N, Mhanna L, Droneau S, Plat G, Didier A, Mazieres J, Hermant C (2016). Techniques of endoscopic airway tumor treatment. Journal of Thoracic Disease.

[ref-10] Kashyap S, Mohapatra PR, Saini V (2003). Endobronchial tuberculosis. Indian Journal of Chest Diseases and Allied Sciences.

[ref-11] Korkmaz Ekren P, Basarik Aydogan B, Gurgun A, Tasbakan MS, Bacakoglu F, Nava S (2016). Can fiberoptic bronchoscopy be applied to critically ill patients treated with noninvasive ventilation for acute respiratory distress syndrome? Prospective observational study. BMC Pulmonary Medicine.

[ref-12] Lin CY, Chung FT (2016). Central airway tumors: interventional bronchoscopy in diagnosis and management. Journal of Thoracic Disease.

[ref-13] Mahmood K, Wahidi MM, Thomas S, Argento AC, Ninan NA, Smathers EC, Shofer SL (2015). Therapeutic bronchoscopy improves spirometry, quality of life, and survival in central airway obstruction. Respiration.

[ref-14] Murgu SD, Egressy K, Laxmanan B, Doblare G, Ortiz-Comino R, Hogarth DK (2016). Central airway obstruction: benign strictures, tracheobronchomalacia, and malignancy-related obstruction. Chest.

[ref-15] Murgu SD, Pecson J, Colt HG (2010). Bronchoscopy during noninvasive ventilation: indications and technique. Respiratory Care.

[ref-16] Nie XM, Cai G, Shen X, Yao XP, Zhao LJ, Huang Y, Han YP, Bai C, Li Q (2012). Bronchoscopy in some tertiary grade A hospitals in China: two years’ development. Chinese Medical Journal.

[ref-17] Ost DE, Ernst A, Grosu HB, Lei X, Diaz-Mendoza J, Slade M, Gildea TR, Machuzak M, Jimenez CA, Toth J, Kovitz KL, Ray C, Greenhill S, Casal RF, Almeida FA, Wahidi M, Eapen GA, Yarmus LB, Morice RC, Benzaquen S, Tremblay A, Simoff M, Registry AB (2015). Complications following therapeutic bronchoscopy for malignant central airway obstruction: results of the AQuIRE registry. Chest.

[ref-18] Payne CB, Goyal PC, Gupta SC (1986). Effects of transoral and transnasal fiberoptic bronchoscopy on oxygenation and cardiac rhythm. Endoscopy.

[ref-19] Sakr L, Dutau H (2010). Massive hemoptysis: an update on the role of bronchoscopy in diagnosis and management. Respiration.

[ref-20] Smith LS, Schillaci RF, Sarlin RF (1987). Endobronchial tuberculosis. Serial fiberoptic bronchoscopy and natural history. Chest.

[ref-21] Vorasubin N, Vira D, Jamal N, Chhetri DK (2014). Airway management and endoscopic treatment of subglottic and tracheal stenosis: the laryngeal mask airway technique. Annals of Otology, Rhinology and Laryngology.

[ref-22] Zhou GW, Zhang W, Dong YC, Huang HD, Hu C, Sun J, Jin F, Gu Y, Li Q, Li S (2016). Flexible bronchoscopy-induced massive bleeding: a 12-year multicentre retrospective cohort study. Respirology.

[ref-23] Zias N, Chroneou A, Gonzalez AV, Gray AW, Lamb CR, Riker DR, Beamis JF (2009). Changing patterns in interventional bronchoscopy. Respirology.

